# Selective Generation of Dopaminergic Precursors from Mouse Fibroblasts by Direct Lineage Conversion

**DOI:** 10.1038/srep12622

**Published:** 2015-07-30

**Authors:** Changhai Tian, Yuju Li, Yunlong Huang, Yongxiang Wang, Dapeng Chen, Jinxu Liu, Xiaobei Deng, Lijun Sun, Kristi Anderson, Xinrui Qi, Yulong Li, R. Lee Mosley, Xiangmei Chen, Jian Huang, Jialin C. Zheng

**Affiliations:** 1 Center for Translational Neurodegeneration and Regenerative Therapy, Shanghai Tenth People’s Hospital affiliated to Tongji University School of Medicine, Shanghai 200072, China; 2 Department of Pharmacology and Experimental Neuroscience; 3 Department of Pathology and Microbiology; 4 Department of Emergency Medicine; 5 University of Nebraska Medical Center, Omaha, NE 68198, USA; 6 Department of Nephrology, Chinese PLA General Hospital, Beijing 100853, P. R. China; 7 Chinese National Human Genome Center at Shanghai, Shanghai 201203, P.R. China

## Abstract

Degeneration of midbrain dopaminergic (DA) neurons is a key pathological event of Parkinson’s disease (PD). Limited adult dopaminergic neurogenesis has led to novel therapeutic strategies such as transplantation of dopaminergic precursors (DPs). However, this strategy is currently restrained by a lack of cell source, the tendency for the DPs to become a glial-restricted state, and the tumor formation after transplantation. Here, we demonstrate the direct conversion of mouse fibroblasts into induced DPs (iDPs) by ectopic expression of Brn2, Sox2 and Foxa2. Besides expression with neural progenitor markers and midbrain genes including Corin, Otx2 and Lmx1a, the iDPs were restricted to dopaminergic neuronal lineage upon differentiation. After transplantation into MPTP-lesioned mice, iDPs differentiated into DA neurons, functionally alleviated the motor deficits, and reduced the loss of striatal DA neuronal axonal termini. Importantly, no iDPs-derived astroctyes and neoplasia were detected in mouse brains after transplantation. We propose that the iDPs from direct reprogramming provides a safe and efficient cell source for PD treatment.

Selective degeneration of functional neurons is a key pathogenic event in many neurodegenerative disorders. Cell replacement through transplantation of stem/progenitor cells represents a particularly promising therapeutic strategy for these diseases. One of the most sought after diseases for cell replacement is Parkinson’s disease (PD), which is a prototypical illness characterized by the loss of dopaminergic (DA) neurons in the substantia nigra pars compacta (SNpc) and decreased DA innervation in the striatum^1^,^2^. Recent breakthroughs in stem cell biology have established the feasibility of directly reprogramming cells of one lineage into another, e.g. neurons, by introducing crucial cell-fate determinants^3^,^4^. As a result of those advances, induced neurons^5^,^6^,^7^,^8^,^9^ and induced neural stem cells (iNPCs)^10^,^11^,^12^,^13^ have been successfully generated, and studies show that these cells hold therapeutic benefits. Direct reprogramming represents an important direction to obtain safe and less controversial cell sources for PD treatment. The low yield and non-proliferative nature of dopaminergic neurons derived from direct reprogramming limits broad application. Multipotent neural stem/progenitor cells (NSCs/NPCs), including iNPCs that give rise to all types of neural cells, may increase the yield of engraftable cells; however, specific and efficient induction of homogeneous DA neurons from NPCs/iNPCs remains a challenge. NSCs/NPCs often respond poorly to pre-patterning morphogens with low differentiation efficiency for specific neuronal subtypes, and are prone to a glial-restricted state^14^. Moreover, grafted NSCs/NPCs are more likely to terminally differentiate into astrocytes rather than functional neurons in response to injury^15^,^16^. Therefore, dopaminergic neuronal-lineage restricted precursors that hold great potential for DA neuronal differentiation are highly desirable for experimental PD treatment.

Studies from our group as well as others have previously revealed that Brn2/Brn4 and Sox2 are critical for the direct conversion of fibroblasts into induced neural progenitors^9^,^10^,^13^,^17^. Strategically, we and others have been working on the direct reprogramming of somatic cells into region-specific iNPCs as well as subtype-specific iNPCs by expressing defined transcript factors in addition to Brn2/Brn4 and Sox2^10^,^18^. The factors that directly reprogram somatic cells into neuronal lineage-restricted progenitors have been expanded to the combination of Brn2/Brn4 and Sox2 with c-Myc 19. In addition, in the presence of Brn2 and Sox2, FoxG1, a transcription factor that is predominantly expressed in the forebrain, contributes to the acquisition of forebrain identity in iNPCs^10^ (our unpublished data). In contrast, the midbrain DA neurons that originated from the floor plate cells in the mesencephalon are critically regulated by transcriptional determinants such as Foxa2^20^,^21^,^22^. Furthermore, the development of DA neurons depends not only on initial Sonic hedgehog (SHH)/ Fibroblast growth factor 8 (FGF8) and Wnt1 signaling pathway for the dopaminergic progenitors, but also on the cooperation of SHH-Foxa2 and Wnt1-Lmx1α pathways^23^.

We hypothesize that the specification of midbrain identity and dopaminergic neural fate could be achieved by the ectopic expression of Brn2 and Sox2 with Foxa2 during the direct reprogramming of terminally differentiated cells. Here, we demonstrate that the addition of Foxa2 into the reprogramming procedure initiated by Brn2 and Sox2 successfully converts adult mouse skin fibroblasts into neural progenitors with a midbrain identity and selective dopaminergic differentiation potential. As a result, the induced DPs (iDPs) are dopaminergic neuronal-restricted *in vitro* and *in vivo*, and do not form tumors or neural overgrowths in the brain. Moreover, grafted iDPs in the striatum of MPTP mouse model differentiated terminally into DA neurons rather than astrocytes, and functionally alleviate PD symptoms. Furthermore, we engineered L-Myc expression in the iDPs under the control of doxycycline (Dox). As a result, the iDPs can be safely expanded to the desired yield for transplantation, providing a useful cell source for PD treatment.

## Results

### Foxa2 cooperates with Brn2 and Sox2 to convert mouse fibroblasts into neural progenitors with midbrain identity

Our initial studies have shown that skin fibroblasts can be successfully converted into induced neural progenitors (5F-iNPCs) by a set of transcription factors, including Brn2, Sox2, TLX, Bmi1 and c-Myc^13^. However, the dopaminergic differentiation efficiency of 5F-iNPCs in response to SHH/FGF8 stimulation remains low (<5%). The direct reprogramming of somatic cells into region-specific iNPCs as well as subtype-specific iNPCs by overexpression of defined transcript factors has become our strategy. It has been suggested that Foxa2 and Lmx1a are the major mediators of SHH and Wnt1 signaling in midbrain DA neuron development, respectively^23^. The role of these factors in cellular reprogramming remains unclear. We utilized a Tet-On/Off system to express Foxa2 and Lmx1a in 5F-iNPCs. The cells that successfully incorporated Foxa2 and Lmx1a genes were further screened with the addition of antibiotics puromycin and neomycin in the culture (Fig. S1-A). In the presence of Dox, the ectopic expressions of Foxa2 and Lmx1a were induced (Fig. S1-B). As expected, both Foxa2- and Lmx1a-expression significantly reduced the proliferation of 5F-iNPCs (Fig. S1-C). Furthermore, the differentiation-related genes, including Ngn2 and Gpm6a for neuronal lineage and Glast for glial lineage, were significantly up-regulated. In contrast, the neural stem cell makers, such as CD133 and Nestin, were down-regulated (Fig. S1-D). Interestingly, the expressions of tyrosine hydroxylase (TH) and several key factors responsible for midbrain dopaminergic neuron development in 5F-iNPCs were dramatically increased in response to Foxa2 and Lmx1a overexpression (Fig. S1-E), suggesting that both Foxa2 and Lmx1a play a positive role in the determination of DA neuronal fate. The effect of Foxa2 and Lmx1a on DA neuronal fate was further confirmed by immunocytochemistry. Foxa2 and Lmx1a overexpression did not affect astrocyte differentiation and neuronal maturation (Fig.S2-A). Under neuronal differentiation conditions with SHH/FGF8, the population of TH^+^ cells was significantly increased following the overexpression of Foxa2 and Lmx1a (Fig.S2-B, left panel). The percentage of TH^+^ cells to total MAP2^+^ cells was about 10-fold higher than those 5F-iNPCs without Foxa2 and Lmx1a overexpression (Fig.S2-B, right panel). Together, these data suggested that Foxa2 may cooperate with Lmx1a to promote the mesencephalic dopaminergic neuron identity in iNPCs, without affecting the neuronal maturation and astrogliogenesis.

**Figure 1:**
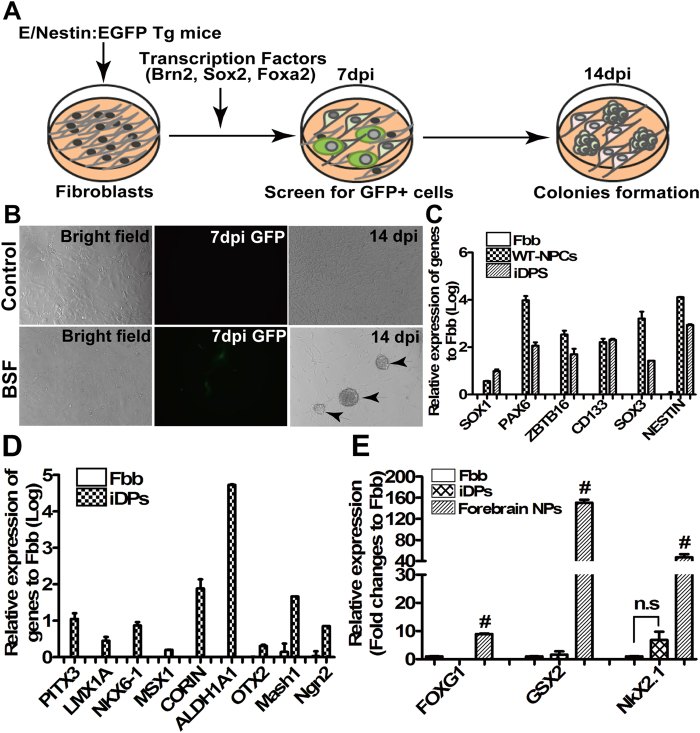
Generation of induced dopaminergic precursors from mouse skin fibroblasts by defined factors. Schematic procedure drawn by Changhai Tian for iDP generation by ectopic overexpression of three transcription factors (Brn2, Sox2 and Foxa2, BSF) (**A)**. Kinetics of iDP generation monitored by GFP occurrence at 7 dpi and clone formation at 14 dpi (indicated by arrows) (**B**). The expression of a specific set of neural progenitor marker genes (**C**), ventral mesencephalon (VM) related genes (**D**), and telencephalon related genes (**E**) by real-time RT-PCR analysis, and GAPDH-specific primer pairs were used for internal control. Fibroblasts (Fbb) are served as negative control, and primary neural progenitors (WT-NPCs) and forebrain neural progenitors (NPs) served as positive controls. #denotes *p* < 0.05 compared to Fbb and iDPs.

Although the specification and maturation of mesencephalic floor plate-originated midbrain DA neurons are primarily regulated by both Foxa2 and Lmx1a/b^24^,^25^, recent evidence has demonstrated that Foxa2 can positively regulate Lmx1a/b and inhibit the expression of Nkx2.2 in neural progenitors^26^. This indicates that Foxa2 alone may suffice for the specification of mesencephalic dopaminergic progenitor identity. We examined this idea by determining the role of Foxa2, along with two known reprogramming factors Brn2 and Sox2, in the direct conversion of somatic cells into neural progenitors. We isolated and cultured adult dermal fibroblasts (SF) from Nestin-EGFP transgenic mice (E/Nestin: EGFP). The fibroblasts were infected with retroviruses encoding Brn2, Sox2 and Foxa2 (BSF) following a schematic procedure (Fig. 1A). The kinetics of induced neural progenitors was monitored with the EGFP in the culture. Seven days after retroviral infections, we observed EGFP positive cells in culture, and at 14 days post-infection, we observed colony formation (Fig. 1B). Seven colonies were obtained and only two were confirmed to be expandable clones after subculture. Both of the expandable clones showed identical properties (data not shown). Compared to the primary cortical neural progenitor cells (WT-NPCs), the resulting cells (named iDPs) retained high expression levels of Foxa2, Brn2 and Sox2 (Fig. S3). Meanwhile, endogenous Foxa2, Brn2 and Sox2 genes expression was also up-regulated, suggesting that the endogenous gene network had been initiated. We characterized the iDPs through the expression levels of several key neural progenitor marker genes in skin fibroblasts (Fbb), WT-NPCs and iDPs by real-time RT-PCR analysis (for sequences of the primer pairs, see Table S1). The iDPs expressed high levels of neural progenitor markers, including Sox1, PAX6, ZBTB16, Sox3, CD133 and Nestin (Fig. 1C). Importantly, the iDPs also expressed high levels of Aldh1A1, Corin (Lrp4), Lmx1a, Msx1, Ngn2, Otx2, Mash1, Pitx3 and Nkx6.1 (Fig. 1D). These genes were previously reported to specifically expressed in dopaminergic neuron proliferative progenitor cells^20^,^23^,^27^,^28^. In contrast, unlike the primary forebrain neural progenitors (NPs), the iDPs expressed the minimum levels of FoxG1, GSX2, and Nkx2.1 (Fig. 1E), indicative of a forebrain identity^10^,^29^. These results suggest that fibroblasts could acquire the mesencephalic regional identity and dopaminergic neural fate through the forced expression of transcription factors Brn2, Sox2, and Foxa2.

### iDPs express specific dopaminergic progenitor markers and possess dopaminergic neuronal-restricted differentiation potential

**Figure 2:**
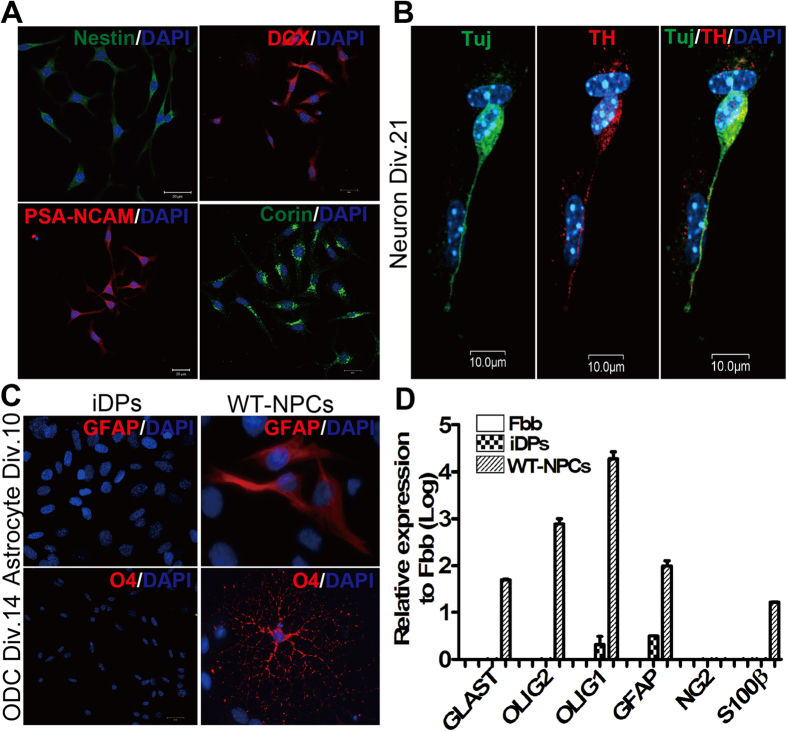
Characterizations of iDPs. iDPs cultures were subjected to immunostaining for Nestin (green), Corin (green), DCX and PSA-NCAM (red), and DAPI (blue) (**A**). iDPs cultured on PLO/laminin-coated coverslips were subjected to dopaminergic neuronal differentiation in the presence of SHH and FGF8, and then immunostaining was performed with antibodies against βIII-Tubulin (green) and Tyrosine hydroxylase (TH, red), and nuclear staining with DAPI (blue) (**B**). mRNAs were collected from Fbb, WT-NPCs and iDPs, and then subjected to real-time RT-PCR analysis with primers specific for glial-lineage identity (*see* Table S1),and GAPDH-specific primer pairs were used for internal control (**C**). WT-NPCs and iDPs cultured on coated coverslips were differentiated with astrocyte medium for 10 days and oligodendrocyte medium for 14 days, respectively, and then subjected to immunostaining with GFAP and O4 (red), and nuclear staining with DAPI (blue) (**D**). (Scale bars: 20 μm).

Ploysialic acid-neural cell adhesion molecule (PSA-NCAM), doublecortin (DCX) and Corin (Lrp4) have been used to identify and isolate neuronal-restricted precursors from ESC-derived neural derivatives^28^,^30^,^31^. We characterized the iDPs through the immunostaining of cells with specific antibodies against PSA-NCAM, DCX and Corin, and observed that the iDPs specifically expressed all three of the marker genes in addition to the NPC marker gene Nestin (Fig. 2A). This suggests that the iDPs are dopaminergic neuron-restricted precursors. We further validated whether the iDPs are dopaminergic neuronal lineage-restricted by differentiating the iDPs into neurons in the presence of SHH and FGF8 stimulation followed by the treatment of BDNF, GDNF and ascorbic acid (AA) for neuronal maturation. Separately, astrocyte and oligodendrocyte differentiation were induced by 10% serum and PDGF/forskolin, respectively. Our results demonstrated that all βIII-tubulin positive neurons derived from iDPs were TH positive (Fig. 2B), suggesting dopaminergic neuronal lineage-restricted fate for the iDPs. In contrast, no GFAP and O4 positive cells were observed after astrocyte and oligodendrocyte differentiation, indicating that the iDPs had committed to the neuronal lineage (Fig. 2C). As a positive control, when WT-NPCs were put in the same astrocyte and oliogodendrocyte differentiation conditions, astrocyte and oliogodendrocyte developed (Fig. 2C). Unlike WT-NPCs, the iDPs also expressed much lower levels of glial-lineage related genes, such as Glast, Olig1/2, NG2, GFAP and S100β (Fig. 2D). Since these genes are critical regulators for glial-lineage development, the low expression profile of these genes further validated that the iDPs had given up their glia differentiation potentials and committed to a neuronal fate.

**Figure 3:**
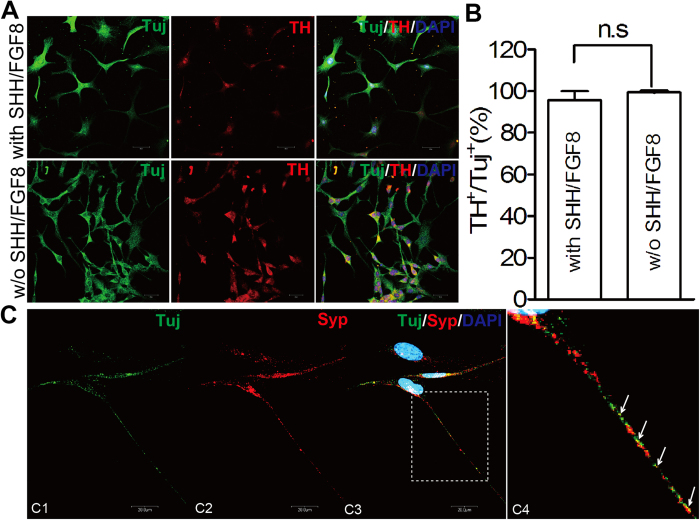
iDPs possess mesencephalic regional identity and differentiate into DA neurons independent of SHH and FGF8 signaling pathway. iDPs were treated with SHH and FGF8 for 6 days, and then terminally differentiated in the presence of BDNF, GDNF, IGF1, TGF-β3, dbcAMP and ascorbic acid for another 3 weeks (A*, upper panel*). Alternatively, iDPs were directly terminally differentiated in the presence of BDNF, GDNF, IGF1, TGF-β3, dbcAMP and ascorbic acid for another 3 weeks (A*, lower panel*). Cells were fixed and then subjected to immunostaining with βIII-Tubulin (green) and Tyrosine hydroxylase (TH, red), and nuclear staining with DAPI (blue) (**A**). The percentage of TH^+^ neurons of total neurons (Tuj^+^) differentiated from iDPs was shown. No significant difference (n.s) was observed (**B**). After differentiation, MAP2^+^ neurons (green) from iDPs were closely associated with presynaptic puncta labeled by synaptophysin (red). Arrows in C4, a large magnification image of box area in C3 shows the presynaptic puncta. (Scale bars: 20 μm)

**Figure 4:**
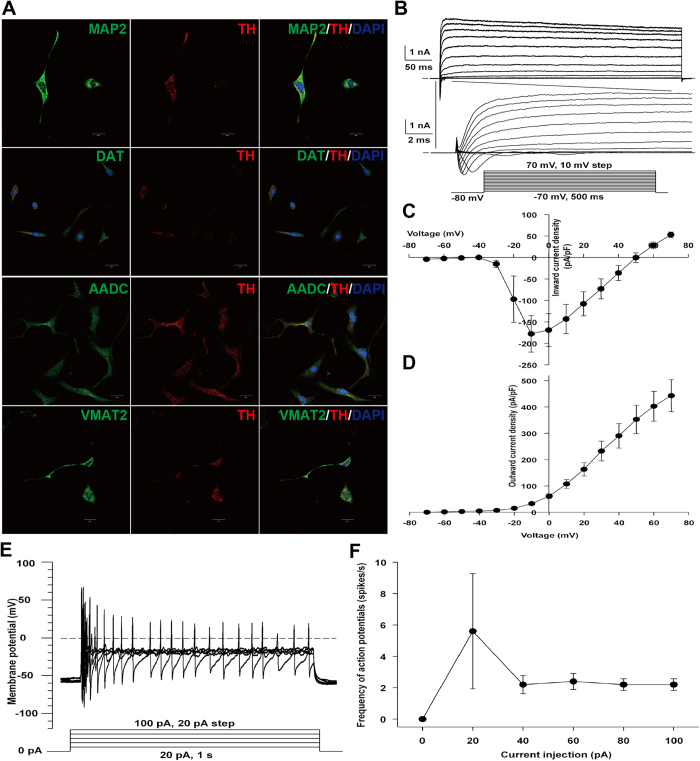
DA neurons derived from iDPs expressed mature neuron markers and exhibited electrophysiological properties. iDPs cultured on PLO/laminin-coated coverslips were terminally differentiated in the presence of BDNF, GDNF, IGF1, TGF-β3, dbcAMP and ascorbic acid for 3 weeks, and then subjected to immunostaining with mature DA neuron markers, including MAP2, TH, AADC, DAT and VMAT2 antibodies, and nuclear staining with DAPI (blue) (**A**). Cells were hyperpolarized to −80 mV for 500 ms before applying depolarizing pulses to elicit inward Na^+^ and outward K^+^ currents (**B**), statistic results showed voltage-dependent inward Na^+^ and outward K^+^ currents (**C**,**D**). The representative traces of membrane potential changes and action potentials elicited by step-current injections (whole-cell recording, current-clamp mode) generated by DA neurons after 3 weeks of iDP differentiation (**E**), and statistic result of action potentials was presented in F (n = 5). (Scale bars: 20 μm)

Next, we tested the differentiation efficiency of iDPs into dopaminergic neurons. We adapted a previously described neuronal differentiation protocol^14^ and found that more than 90% of the Tuj^+^ neurons are TH^+^ dopaminergic neurons (Fig. 3A). Surprisingly, the efficiency for the iDPs to differentiate into dopaminergic neurons remained high in the absence of the pre-patterning morphogens SHH and FGF8 (Fig. 3B), strongly suggesting the dopaminergic neural fate of iDPs. The iDP-derived neurons also expressed synaptophysin involved in neurotransmitter exocytosis and neuroendocrine^32^. The synaptophysin staining appeared positive in cells of typical neuronal morphology. In contrast, an adjacent cell of different morphology appeared negative for synaptophysin, suggesting a specific staining for synaptophysin (Fig. 3, C1–3). Furthermore, the synaptophysin had punctate distribution (Fig. 3, C4), indicative of the synaptic formation *in vitro*. Notably, the iDP-derived DA neurons also exhibited immunoreactivities for aromatic L-amino acid decarboxylase (AADC), the dopamine transporter (DAT) and the brain-specific isoform of the vesicular monoamine transporter (VMAT2) (Fig. 4A), which are three major functionally relevant proteins in dopaminergic neurons. Importantly, the DA neurons derived from iDPs exhibited functional membrane properties of mature neuron. In voltage clamp mode, voltage-gated K^+^ currents and Na^+^ currents can be evoked from a holding potential of −80 mV to test potentials range from −70 mV to + 70 mV in 10-mV steps (Fig. 4B–D). In current clamp mode, action potentials can be evoked in all iDPs tested by injection currents from 20 pA to 100 pA in 20-pA steps (Fig. 4E,F, n = 5). Those results strongly suggest that iDPs can effectively differentiate and generate mature and functional DA neurons.

### Conditional expression of L-Myc induces self-renewal of iDPs

**Figure 5:**
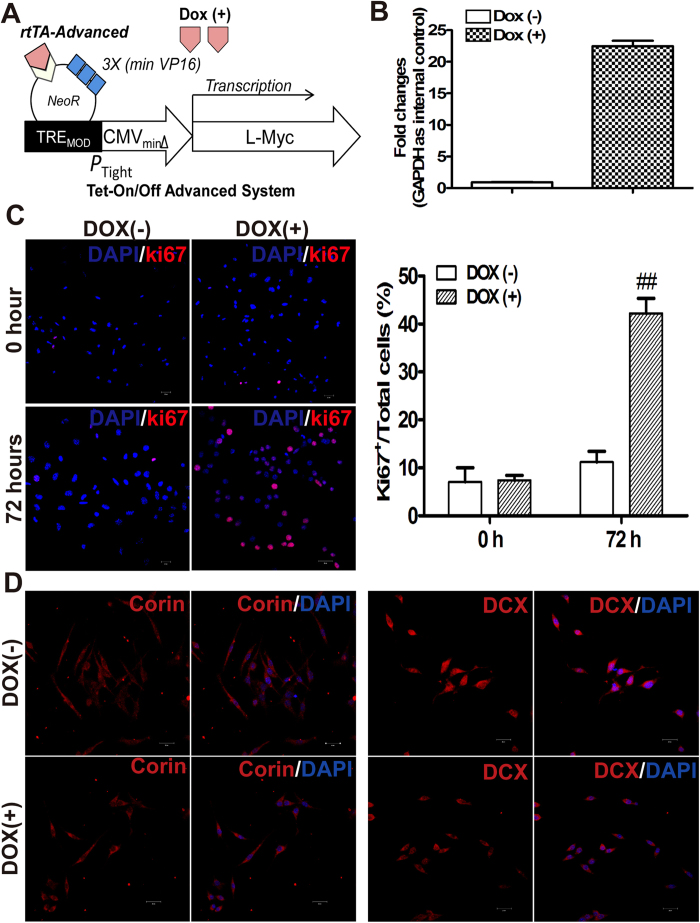
Proliferation and self-renewal of iDPs were enhanced by conditional expression of L-Myc. Schematic diagram of Tet-On/Off advanced system for L-Myc overexpression in iDPs (**A**). Dox-regulated expression of myc (isoform L-myc) in iDPs by real-time RT-PCR analysis with primers specific for L-myc (*see* table S1), and GAPDH-specific primer pairs were used for internal control (**B**). Immunofluorescence staining for Ki67 (red) of cells grown in Dox (+) or Dox (− media for 72 h, and nuclei were stained with DAPI (blue) (**C***, left panel*). Results are shown as the percentage of Ki67^+^ cells of total cells (**C**, *right panel*). ##denotes *p* < 0.001 compared to cells cultured with Dox (−) medium for 72 h. iDPs cultured with Dox (+) or Dox (−) media, respectively, were subjected to immunostaining with Corin and DCX (red) antibodies, and nuclear staining with DAPI (blue) (**D**). (Scale bars: 20 μm)

During the generation of iDPs, we observed that iDPs exhibited limited ability of self-renewal and clonogenicity, restricting cell expansion required for cell transplantation. We overcame this disadvantage by utilizing a Tet-On/Off system to transduce L-Myc, which is a transformation-deficient, safe and efficient approach that enhances self-renewal^33^,^34^ in iDPs. The conditional expression of L-Myc was under the control of doxycycline (Dox) (Fig. 5A,B). The proportion of Ki67 positive cells significantly increased after Dox treatment for 72 hours (Fig. 5C), whereas the expression levels of DP marker genes Corin and DCX were not affected by the Dox treatment. These results confirmed that using L-Myc to induce self-renewal in iDPs is a safe, effective and efficient strategy for cell expansion intended for cell transplantation.

### Grafted iDPs survive, differentiate into dopaminergic lineage and functionally alleviate motor deficits in a MPTP mouse model of PD

**Figure 6:**
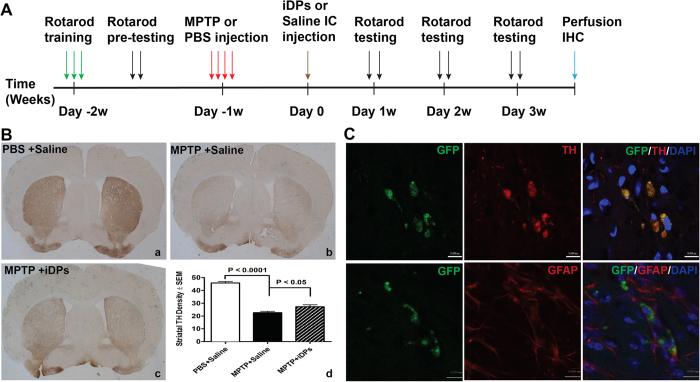
Grafted iDPs survive, differentiate into dopaminergic lineage and partially reverse MPTP striatal pathology. (**A**) The training, pretesting, MPTP administration, iDPs inoculation, testing of motor functions, and experimental end point for sacrifice of the mice are shown in the timeline. (**B**) One week after MPTP intoxication, GFP^+^ iDPs were intracranially injected into the striatum of C57BL/6 mice. Free-floating sections of brain specimens were prepared 3 weeks after iDPs engraftment. Representative photomicrographs of striatum after PBS injection (**a**), MPTP intoxication with saline IC injection (**b**), and MPTP with iDPs IC injection (**c**) were shown. Striatal TH^+^ densities of DA termini were quantified and data represent means ± SEM of striatal densities. Four striatal sections from each mouse were used for analysis. N = 10 mice for saline IC injection group; N = 7 mice for MPTP group and MPTP with iDPs IC injection group. (**C**) Survival and differentiation of GFP^+^ iDPs following IC injection into the striatum of mice were evaluated 3 weeks post-IC injection. (**D**) The grafted cells were detected by immunostaining with antibodies against GFP, TH and GFAP (red), and nuclear staining with DAPI (blue). (Scale bars: 20 μm).

**Figure 7:**
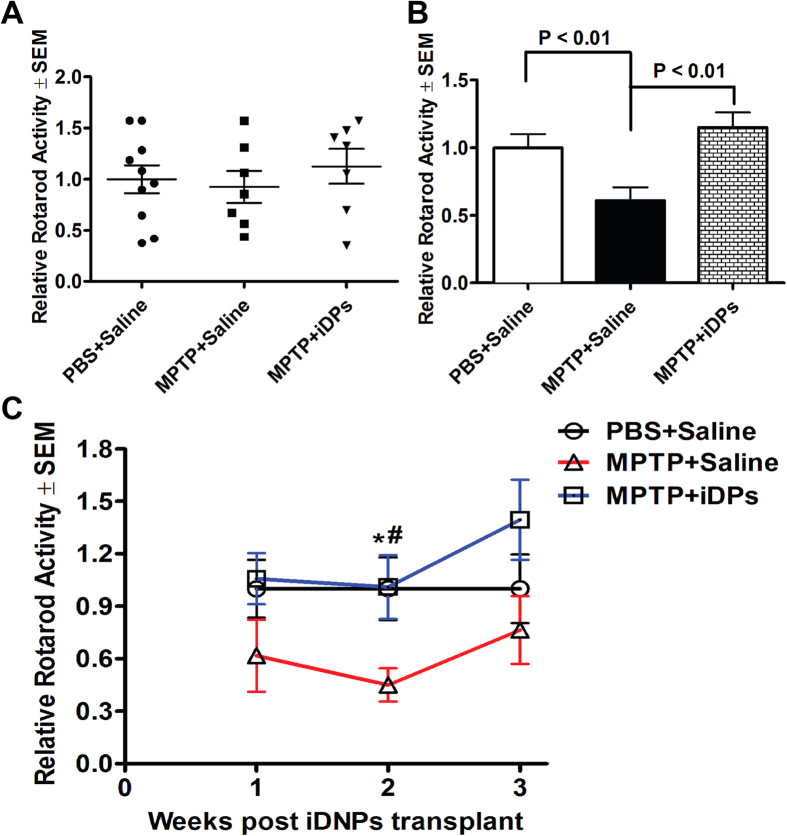
iDPs improve rotarod performance in MPTP-intoxicated mice. Mice were tested twice a week with rotarod motor function tests for 3 weeks following iDPs engraftment. (**A**) Pre-MPTP performance values for all mice group were determined with rotarod. (**B**) Weekly performance values for all mice group at week 2 were shown. (**C**) Weekly performance values for all mice group at week 1–3 were shown. Data represent means ± SEM of each group normalized to baseline rotarod performance. Statistical analysis was performed using two-tailed Student’s t tests. N = 10 mice for saline IC injection group; N = 7 mice for MPTP group and MPTP with iDPs IC injection group.

We tested whether the iDPs with conditional L-Myc expression will be safe, and can survive, terminally differentiate into specific dopaminergic neurons *in vivo*. First, we labeled iDPs with lentiviral vector expressing green fluorescent protein (GFP), and further enriched GFP^+^ cells by the puromycin resistance in vector (Fig. S4-A). GFP^+^ iDPs were then injected into the striatum of a SCID mouse brain as illustrated in Fig. S4-B. At 6 weeks following transplantation, we observed that the grafted GFP^+^ iDP-derived cells were distributed predominantly around the injection site (Fig. S4-C), and the iDP-grafted mice showed no tumor formation at the injection site (Fig. S4-D). These results suggest that engineered iDPs are safe for cell transplantation. To evaluate the functionalities of iDPs *in vivo*, we used a MPTP mouse model of PD as we previously described^35^, and performed cell transplantation in PD mouse model as experimental timeline illustrated in Fig. 6A. The mice were trained with rotarod and two trials were performed before MPTP intoxication to establish baseline performance. At one week after MPTP intoxication, iDPs were engrafted on both side of striatum through intracranial (IC) injection. The mice were monitored for 3 weeks for motor functions and then sacrificed for immunohistochemistry. The densities of TH^+^ DA neuron axonal termini in the striatum were determined by digital image analysis as previously described^36^. The MPTP treatment significantly decreased the densities of TH^+^ striatal termini, suggesting substantial loss of DA neuronal termini in striatum (Fig. 6B). Densitometric analysis of TH^+^ striatal termini by week 3 showed 50% loss of TH^+^ striatal termini. However, engraftment of MPTP mice with iDPs increased striatal termini densities by 20% (40% total loss, Fig. 6B). Interestingly, a majority of the iDP-derived cells were Tuj and TH double positive, but not GFAP positive cells (Fig. 6C), suggesting that the grafted cells survived *in vivo* for three weeks, and were preferentially differentiated into DA neurons but not astrocytes. Next, we evaluated the effect of iDPs engraftment on motor function of the MPTP mice. All mice group had similar baseline locomotor performance during pre-MPTP testing (Fig. 7A). Rotarod testing of MPTP mice by week 2 showed 55% reduction of locomotor functions. Engraftment of MPTP mice with iDPs increased rotarod performance (Fig. 7B,C). By week 3 (4 weeks after MPTP intoxication), the MPTP mice showed 24% motor deficits compared with PBS group. The iDPs engraftment group had beneficial locomotor effects but no significant recovery of motor deficits (P = 0.059, Fig. 7C). Together, these data suggest that iDPs engraftment after the development of MPTP lesion increase motor function in mice.

## Discussion

Stem cell-based therapy holds a promising future for the treatment of neurodegenerative disorders, particularly in the case of PD. To achieve cell-based therapy for PD, significant efforts have been made to differentiate human embryonic stem cells (ESCs) and induced pluripotent stem cells (iPSCs) to midbrain DA neurons following morphogenic stimulation^37^,^38^,^39^,^40^. Recently, direct conversion of cells of one lineage into another has served as an alternative strategy for engraftable cell sources. As a result, induced DA neurons^7^,^41^ and induced neural stem/progenitor cells (iNPCs)^10^,^11^,^12^,^13^ were generated and showed promise for DA neuron generation. However, the differentiation efficiency, safety, and the specificity of these cells remain a challenge. In the present study, we reported a novel cocktail of three transcription factors (Brn2, Sox2 and Foxa2) that successfully converted mouse skin fibroblasts into neural progenitors. The resulting iDPs were dopaminergic neuronal-restricted and independent of the pre-patterning by SHH/FGF8 (Figs 1,2 and 3). Most importantly, DA neurons derived from iDPs expressed the mature DA neuron markers *in vitro,* exhibited electrophysiological properties of mature DA neurons (Fig. 4), and survived *in vivo* for 6 weeks without tumor formation (Fig. S4), suggesting the iDPs can serve as a safe and efficient cell source for PD treatment.

Previous studies have employed Brn2/Brn4 and Sox2 as neural fate determinants for direct reprogramming of neural progenitor cells from fibroblasts^10^,^13^,^17^,^19^, but additional factors may be required to achieve the regional specification and neuronal-lineage restriction^10^,^19^. Foxa2 and Lmx1a appear to be critical factors that support the regulatory networks required for midbrain DA specification and the transcriptional control of midbrain dopaminergic development. To selectively generate the dopaminergic precursors, we decided to express Foxa2 and Lmx1a in 5F-iNPCs previously developed by our laboratory. The expression of Foxa2 and Lmx1a endowed 5F-iNPCs with midbrain identity and dramatically increased the yield of TH^+^ neurons (Fig. S1, Fig. S2), suggesting that these two factors could be used to improve the iNPCs-based cell therapy in PD.

Foxa2 and Lmx1a/b are known to cooperatively regulate the proliferation, specification, and differentiation of midbrain dopaminergic progenitors. More specifically, Foxa2 and Lmx1a/b are key transcription factors downstream of strong morphogens (SHH and Wnt1, respectively) that synergistically control the midbrain dopaminergic differentiation by forming two regulatory loops^23^,^42^. Previous studies on Foxa1/2 predominantly concentrated on the specification of midbrain progenitor identity and their regulatory roles on Lmx1a/b and Helt^26^,^43^,^44^,^45^. Foxa2 expression is restricted to the mesencephalic floor plate where it plays both SHH-dependent and -independent roles in the specification of floor plate from which DA neurons originate^25^,^46^. It is likely that Foxa2 may be a more potent determinant of midbrain DA progenitors than Lmx1a and SHH. However, it is unclear whether Foxa2 could serve as a reprogramming factor favoring the generation of DA neural progenitors. In this study, we have tested novel sets of transcription factors that included Brn2 and Sox2 with one or both of Foxa2 and Lmx1a in the reprogramming of fibroblasts as illustrated in the procedure (Fig. 1A). We observed that all combinations resulted in the clonogenicity at 14 day post-infection (data not shown). However, only the Brn2, Sox2 and Foxa2 set successfully converted fibroblasts into stable and expansible iDPs. The iDPs expressed the specific neural progenitor markers, and also exhibited the midbrain identity characterized by the marker genes for dopaminergic neural progenitor cells, such as Aldh1A1, Corin (Lrp4), Lmx1a, Msx1, Ngn2, Otx2, Mash1, Pitx3 and Nkx6.1 (Fig. 1). Among these markers, Aldh1A1 and Corin have been used to specifically mark DPs in the brain and isolate DPs from ES cell-derivatives^28^,^47^. In addition, DCX and PSA-NCAM have been used to isolate the neuronal lineage-restricted progenitors^24^,^30^,^31^. Otx2 prevents the serotonin fate of the progenitors by repressing Nkx2.2^48^, and Foxa2 negatively regulates Helt to inhibit the GABAergic neuron differentiation in the ventral midbrain^43^,^44^,^45^. Because the reprogramming factors Brn2 and Sox2 could not generate DA-specified iNPCs, our results suggest that Foxa2 can work cooperatively with Brn2 and Sox2 to drive the direct conversion of fibroblasts into iNPCs with midbrain progenitor identity.

Our present study demonstrates that ectopic expression of Foxa2 positively regulates Lmx1a, Otx2 and other key transcription factors responsible for DA neuron development, and negatively regulates transcription factors related to glial cell development (Fig. 2). Thus, the resulting iDPs are committed to the dopaminergic lineage with minimum glial cells differentiation potential (Fig. 2). Terminal differentiation in the presence of BDNF, GDNF and AA could steer the iDPs into mature and functional DA neurons independent of SHH/FGF8 stimulation (Figs 3 and 4). This role of Foxa2 is consistent with a previous report that shows Foxa2 can execute the midbrain floor plate program via SHH-independent and -dependent mechanisms^46^.

*In vivo* grafted NSCs/NPCs often terminally differentiate into astrocytes rather than functional neurons in response to injury^15^,^16^. Our strategy in engineering iDPs with L-Myc expression using a Tet-on system (Fig. 5) is a confirmation of the way to efficiently, safely and practically control the self-renewal of iDPs^33^,^34^. When the iDPs were transplanted into the striatum of MPTP PD mouse model following the timeline illustrated in Fig. 6A, we observed that MPTP injection damaged TH^+^ neurons in the striatum, whereas grafted iDPs significantly recovered the striatal TH density after cell transplantation, and very few of them terminally differentiated into astrocytes. Indeed, the majority of cells differentiated into DA neurons at 6 weeks post-transplantation, and no tumor or cell outgrowths were observed, and the partially recovery of motor function in PD mouse model after cell transplantation further suggest that the iDPs may serve as a useful cell source for PD treatment.

Collectively, we demonstrate that Brn2, Sox2 and Foxa2 can successfully convert mouse skin fibroblasts into induced neural progenitors, and Foxa2 confers the induced neural progenitors with the specification of midbrain identity and DA lineage. The success of this work could also provide an important foundation for a cellular model to investigate the pathogenesis of PD when patient skin fibroblasts are reprogrammed into iDPs with Brn2, Sox2 and Foxa2 *in vitro*. Finally, the managed expansion of the engineered iDPs further provides a promising therapeutic cell source for PD.

## Methods

### Cell preparation, retroviral packaging, infection and direct reprogramming

Mouse skin fibroblasts were isolated from adult Nestin-EGFP transgenic mice (kindly provided by Richard J Miller from Northwestern University, Chicago, IL) aged 5.5–7.0 weeks as previously described^13^, with approval of the University of Nebraska Medical Center Institutional Animal Care and Use Committee and following National Institutes of Health (NIH) ethical guidelines. Fibroblasts were cultured in Dulbecco’s modified Eagle’s medium supplemented with 10% FBS, 1 × Non-Essential Amino Acid, 100 U/ml penicillin, 100 μg/ml streptomycin at 37 °C in a 5% CO2 humidified atmosphere. Fibroblasts were used within passage 2–5 to avoid replicative senescence. Mouse Brn2 ORF (EcoRI + Not I), Foxa2 ORF (BamHI + Xho I) were cloned into pMXs-retroviral vectors (Cellbiolabs, RTV-010), and pMXs-Sox2 was purchased from Addgene (Plasmid #13367). Retroviruses (pMXs) were generated with Plat-E packaging cells as previously described^49^. In brief, Plat-E cells were seeded at 3.6 × 10^6^ cells per 100-mm dish. 24 hours after seeding, 15 μg DNA of pMXs-based retroviral vectors encoding Sox2, Brn2 and Foxa2 were introduced into Plat-E cells using 15 μl of Lipofectamine™ LTX transfection reagent (Invitrogen). The medium was replaced with 5 ml of DMEM containing 5% FBS 24h after transfection. Fibroblasts from Nestin-EGFP transgenic mice were seeded at 2 × 10^5^ cells per 35-mm dish. At 48h and 72h post-transfection, virus-containing supernatants from these Plat-E cultures were recovered and filtered through a 0.45-μm cellulose acetate filter. Equal volumes of the supernatants were mixed and supplemented with 10 μg/ml polybrene. Cells were incubated in virus/polybrene-containing supernatants overnight. The medium was changed 3 days after infection to NeuroCult® NSC Basal (Stem Cell Technologies, Inc.,Vancouver, BC V5Z 1B3, Canada) Medium supplemented with NeuroCult® NSC Proliferation Supplements (Stem Cell Technologies, Inc.), 20 ng/ml basic fibroblast growth factor (bFGF, BioWalkersville), and 20 ng/ml epidermal growth factor (EGF, BioWalkersville). After 9–14 days, the predicted iDP colonies were monitored by fluorescence microscope.

### Quantitative Real-Time RT-PCR

Total mRNA was isolated with TRIzol Reagent (Invitrogen) and RNeasy Mini Kit (QIAGEN Inc., Valencia, CA) using the manufacturer’s recommendations. The reverse transcription was performed using Transcription 1^st^ Strand cDNA Synthesis Kit (Roche, USA). The RT-PCR analyses for the detection of neural stem cell-specific mRNAs were performed using SYBR^®^ Select Master Mix (Life Technologies, Los Angeles, CA) with 0.5 μl of cDNA, corresponding to 1 μg of total RNA in a 15 μl final volume, 1.5 μl H_2_O, 7.5 μl SYBR Green, 5.5 μl oligonucleotide primer pairs (synthesized at Fisher) at 10 μM (See Table S1). PCR program: 1. 50 °C for 2 min, 2. 95 °C for 2 min; 3. 95 °C for 15 sec, 4. specific annealing temperature for 15 sec, 5. 72 °C for 1min. Steps 2–4 were repeated 40 times. All samples were amplified in triplicate and the mean was used for further analysis.

### Immunocytochemistry

The cultured cells were fixed in 4% formaldehyde for 20 min at room temperature and then washed with PBS for three times. The fixed cells were permeabilized with 0.2% Triton X-100 in PBS for 10 min, and blocked with 2% BSA in PBS for 1 h at room temperature. Cells were incubated with primary antibodies as listed in supplementary material Table S1 overnight, and then washed with PBS for three times and incubated for 2 h at room temperature with secondary antibodies (See Table S2). Fluorescent images were obtained using a Zeiss 710 Confocal Laser Scanning Microscope (Carl Zeiss, Oberkochen, Germany).

### Differentiation

For astrocyte differentiation, the iDP culture medium was replaced by DMEM/F-12 with 10% FBS, and cultured for 7 days, with the medium changed every other day. For oligodendrocyte differentiation, iDPs were cultivated in DMEM/F12 with 1 × N2, 10 ng/ml PDGF (R&D Systems, Minneapolis, MN), 10 ng/ml FGF-2 and 10 μM forskolin (Sigma-Aldrich, Saint Louis, MO) for 4 days. Afterwards, PDGF and forskolin were replaced by 30 ng/ml 3, 3, 5-triiodothyronine (T3) hormone and 200 mM ascorbic acid (all from Sigma-Aldrich, Saint Louis, MO) for another 7 days. For neuronal differentiation, SHH/FGF8 independent protocol: iDPs were plated on poly-L-Ornithine/laminin-coated coverslips in 24-well plate with DMEM/F12 supplemented with 1 × N2, 1 × B27, 1.0 mM Glutamax, 0.11 mM β-mercaptoethanol, 1.0 mM dibutyrylcAMP (Sigma), 0.2 mM ascorbic acid (Sigma), 10 ng/mL brain-derived neurotrophic factor (BDNF) (Peprotech), and 10 ng/mL glial cell line-derived neurotrophic factor (GDNF) (Peprotech) for 4 weeks. The medium was changed every 3-4 days. Unless otherwise indicated, all reagents were purchased from Invitrogen (Carlsbad, CA, USA); SHH/FGF8 dependent protocol: Cells were plated on poly-L-Ornithine/laminin-coated coverslips in 24-well plate with DMEM/F12 containing with 1 × N2, 10 ng/ml bFGF (Peprotech), 100 ng/ml SHH (Peprotech), 100 ng/ml FGF8 (Peprotech) for 6 days, and then switched to DMEM/F12 containing 1 × N2, 1 × B27, 1.0 mM Glutamax, 0.11 mM β-mercaptoethanol, 1.0 mM dibutyrylcAMP (Sigma), 0.2 mM ascorbic acid (Sigma), 10 ng/mL brain-derived neurotrophic factor (BDNF) (Peprotech), and 10 ng/mL glial cell line-derived neurotrophic factor (GDNF) (Peprotech) for another 4 weeks, and the medium was changed every 3–4 days.

### Retroviral Infection, Isolation, and Expansion of iDPs

L-Myc were cloned into the Tet-All retroviral-vector, which contains both reverse tetracycline-controlled transactivator (rtTA) and tetracycline response element (TRE) promoter. The original backbones for the Tet-All vector were pRetroX-Tight and pRetro-rtAT-Advanced from Clontech Laboratories (Mountain View, CA, USA). The retrovirus encoding L-Myc was packaged in Plat-E cells, and iDPs were infected with the virus and screened with neomycin (neo).

### Recording of action potentials and total currents

Action potentials and total currents were recorded by the whole cell patch-clamp technique using Axonpatch 200B patch-clamp amplifier (Axon Instruments, Sunnyvale, CA). pClamp 10.2 program was used for data acquisition. The patch-pipette solution was composed of (in mM): 105 K-aspartate, 20 KCl, 1 CaCl_2_, 5 MgATP, 10 HEPES, 10 EGTA, and 25 Glucose (pH 7.2; 320 mOsm/L). The bath solution consisted of (in mM): 140 NaCl, 5.4 KCl, 0.5 MgCl_2_, 2.5 CaCl_2_, 5.5 HEPES, 11 Glucose, and 10 Sucrose (pH 7.4; 330 mOsm/L). The same pipette and bath solutions were used for action potentials and total currents measurement. Resistance of the patch pipette was 3–5 MΩ. Series resistance of 6–13 MΩ was electronically compensated 80–90%. Action potentials were elicited by a series of 1-s depolarizing currents injection from 20 pA to 100 pA with 20-pA increments. Total currents were evoked from a holding potential of −80 mV by stepping to voltages between −70 and + 70 mV in 10 mV increment for 500 ms. Recorded traces were sampled at 10 kHz and filtered at 5 kHz. All experiments were done at room temperature.

### MPTP intoxication and iDPs engraftment

Adult male 8-week-old C57BL/6J mice were purchased from Jackson Laboratories (Bar Harbor, ME) and housed on a 12:12 h light/dark cycle with ad libitum access to food and water in the animal facilities at the University of Nebraska Medical Center. All animal procedures were conducted according to protocols approved by the Institutional Animal Care and Use Committee at the University of Nebraska Medical Center. Mice were randomly assigned to treatment groups before motor function and behavior training (Fig. 6A). MPTP intoxication was performed as previously described^35^. Briefly, mice received four subcutaneous injections, one every 2 h of either vehicle (phosphate buffered saline (PBS) at 10 ml/kg) or MPTP-HCL (20 mg/kg, free base in PBS; Sigma-Aldrich Co, St. Louis, MO). MPTP handling and safety measures were in accordance with the published guidelines^50^. For iDPs engraftment, at 7 days after MPTP intoxication, mice were anesthetized with Ketamine (120 mg/kg) and xylazine (16 mg/kg) by i.p, placed in a stereotaxic apparatus (Stoelting, Wood Dale, IL, www.stoeltingco.com) for IC injection (Fig. 6A). A linear skin incision was made over the bregma, and two 1-mm burr holes were drilled in the skull 0.2 mm posterior and 3.5 mm lateral to the bregma using a hand-held driller. iDPs (0.25 million per injection) were labeled with GFP expressing retrovirus and injected into the striatum of both hemispheres. Saline was used as a control for iDPs injection. Coordinates for inoculation were set as: 0.22 mm posterior to bregma, 3.25 mm lateral from the Sagittal midline, and a depth of 2.9 mm in vertical line. A Hamilton 10-μl syringe (Fisher) was used for cell injection. Mice were sacrificed 21 days post-injection after intracardiac perfusion and brains were quickly removed. Immunohistochemistry for TH^+^ nerve terminals and quantification were performed in a blinded fashion as previously described^35^,^51^.

### Motor function and behavior tests

Rotarod test was used to evaluate the recovery of MPTP-induced motor deficits by the engrafted iDPs. The apparatus was fitted with a 7-cm diameter rod and was interfaced with automatic timing instrumentation (Rotamex, Columbus Instruments, Inc., Columbus, OH, USA). Mice were habituated and trained to perform on the accelerating rotarod at 2–12 rpm for 5 min for four daily sessions in three consecutive days prior to the administration of MPTP. Twice before MPTP-intoxication, all mice were evaluated at 10 rpm for a maximum of 90 s per trial to obtain a pre-treatment baseline performance. After iDPs engraftment, mice were re-evaluated on the rotarod twice a week for 3 weeks. Weekly post-treatment rotarod performance was calculated as average of two trials and as a ratio relative to animal group’s baseline performance. Scores from MPTP mice with or without iDPs engraftment were normalized to the mean performance of the PBS control group^35^,^51^.

### Statistical Analysis

Data were expressed as means ± SD and statistically evaluated by analysis of variance (ANOVA) followed by the Tukey’s test for paired observations unless specified. Significance was considered as a p value of < 0.05. All assays were performed at least twice, with triplicate samples in each experiment.

## Additional Information

**How to cite this article**: Tian, C. *et al*. Selective Generation of Dopaminergic Precursors from Mouse Fibroblasts by Direct Lineage Conversion. *Sci. Rep*. **5**, 12622; doi: 10.1038/srep12622 (2015).

## Supplementary Material

Supplementary Information
