# Supplementary material for: Selective Generation of Dopaminergic Precursors from Mouse Fibroblasts by Direct Lineage Conversion
Source: Sci Rep. 2015 Jul 30;5:12622. doi: 10.1038/srep12622 (PMC4519786; doi:10.1038/srep12622)
Supplement: Supplementary Information [file srep12622-s1.pdf]

SUPPLEMENARY INFORMATION

Selective Generation of Dopaminergic Precursors from Mouse Fibroblasts by Direct Lineage Conversion

Changhai Tian[1,2,5,#,ξ], Yuju Li[2,5,#], Yunlong Huang[1,2,5,#], Yongxiang Wang[2,5], Dapeng Chen[6], Jinxu Liu[4], Xiaobei Deng[1], Lijun Sun[3,5], Kristi Anderson[2,5], Xinrui Qi[1], Yulong Li[4], R. Lee Mosley[2,5], Xiangmei Chen[6], Jian Huang[7], Jialin C. Zheng[1,2,3,5,ξ]

Supplemental Methods

Cell labeling, Transplantation and Histology iDPs with controllable L-Myc (Tet-On/Off advanced system) were transduced by lentiviruses packaged by pLenti-CMV-GFP-Puro with psPAX2 and pMD2.G (Addgene) in 293T cells using Lipofectamine® LTX Reagent with PLUS™ Reagent (Invitrogen™). Titers of lentiviral preparations were determined using 293T cells and ranged between 10^7–10^8 IFU/ml. iDPs were expanded in NeuroCult® NSC basal medium supplemented with NeuroCult® NSC Proliferation supplements (Stem Cell Technologies, Inc.), 20 ng/ml bFGF (BioWalkersville), and 20 ng/ml EGF (BioWalkersville) and screened by adding Dox (4 μg/ml) and G418 (400 ng/ml). Four-week-old male C.B.-17 SCID mice were purchased from the Charles River Laboratory. All mice were housed in the animal facilities at the University of Nebraska Medical Center. All procedures were conducted according to protocols approved by the Institutional Animal Care and Use Committee (IACUC) of the University of Nebraska Medical Center. Briefly, mice were anesthetized with Ketamine (120 mg/kg) and xylazine (16 mg/kg) by i.p, placed in a stereotaxic apparatus (Stoetling, Wood Dale, IL) for intracranial injection in the left cerebral hemisphere. GFP-expressing iDPs (0.5×10^6/5 μl) were injected with a 10-μl syringe into the striatum of mouse brain. Coordinates for inoculation were set as: 0.5-0.8 mm posterior to bregma, 3.5 mm lateral form the sagittal midline, a depth and angle of 3.6 mm and 35° from the vertical line. Mice were anesthetized and perfused with 4% paraformaldehyde (PFA) 6 weeks after injection, and brains were immediately removed and then placed in 4% PFA for postfixation overnight. After dehydration in 30% sucrose solution, brains were frozen and cut on a cryostat (30 μm), and sections were then scanned using a Leica Confocal Microscope and Metamorph

analysis software (Molecular Devices). In addition, fixed brains were embedded in paraffin, and sections were stained with hematoxylin and eosin (H&E), and images were acquired and analyzed by Ventana's Coreo Au Slider Scanner.

FIG.S1

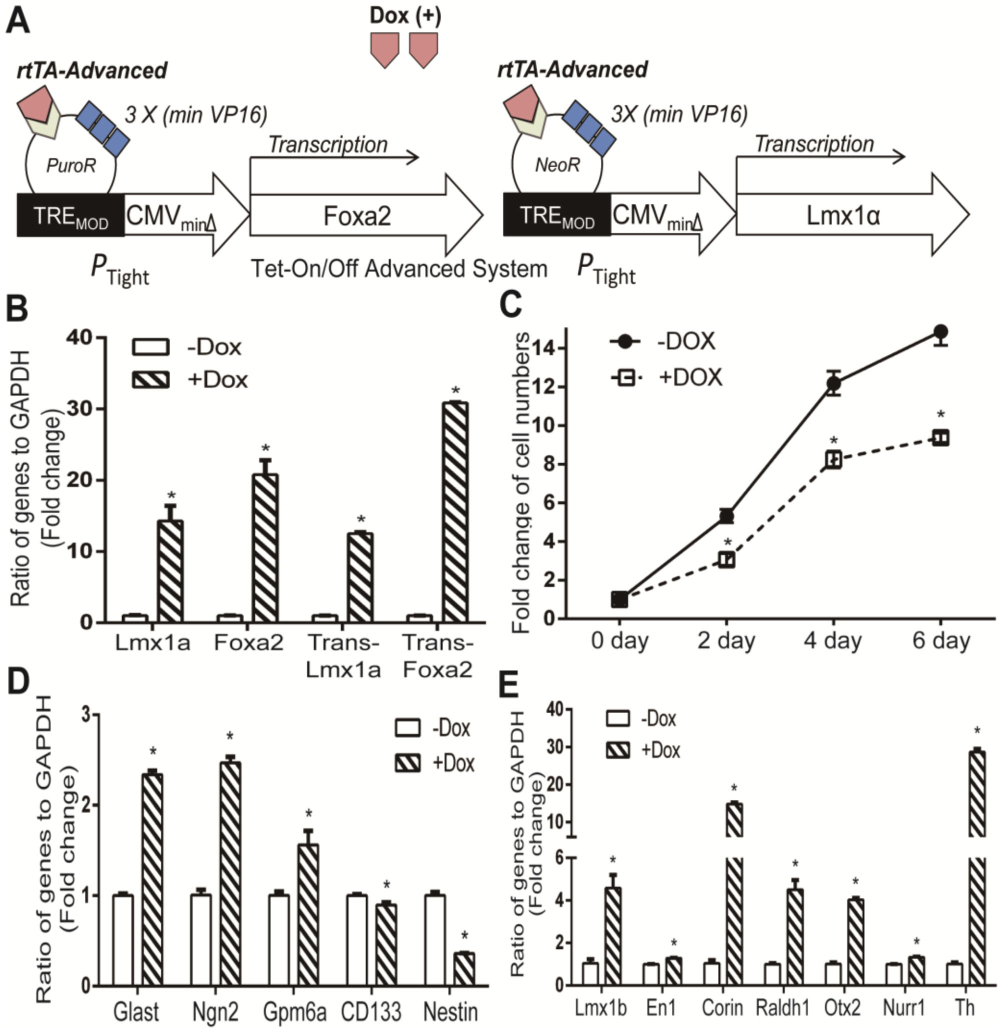

Supplemental figure 1: Ectopic expression of Lmx1a and Foxa2 inhibits the proliferation of 5F-iNPCs and confers the mesencephalic regional identity on 5F-iNPCs

Schematic diagram of Tet-On/Off advanced system for Lmx1α and Foxa2 overexpression in 5F-iNPCs (*A*). Infected 5F-iNPCs were treated with Dox (+) and Dox (-) media for 4 days, and the total and transgene levels of Lmx1a and Foxa2 in 5F-iNPCs were investigated through real-time RT-PCR with specific primers (*see table S1*) (*B*). 1×10^5 5F-iNPCs were cultured in the 6-well plate with or without Dox, respectively, and the

cell numbers were counted at day 2, day 4, and day 6 (*C*). 5F-iNPCs were cultured with or without Dox (1 μg/ml) for 4 days, and mRNA were collected and then subjected to real-time RT-PCR with specific primers for glial lineage, neuronal lineage and dopaminergic neuron-related genes (*D*). GAPDH-specific primer pairs were used for internal control. *denotes $p < 0.05$.

FIG. S2

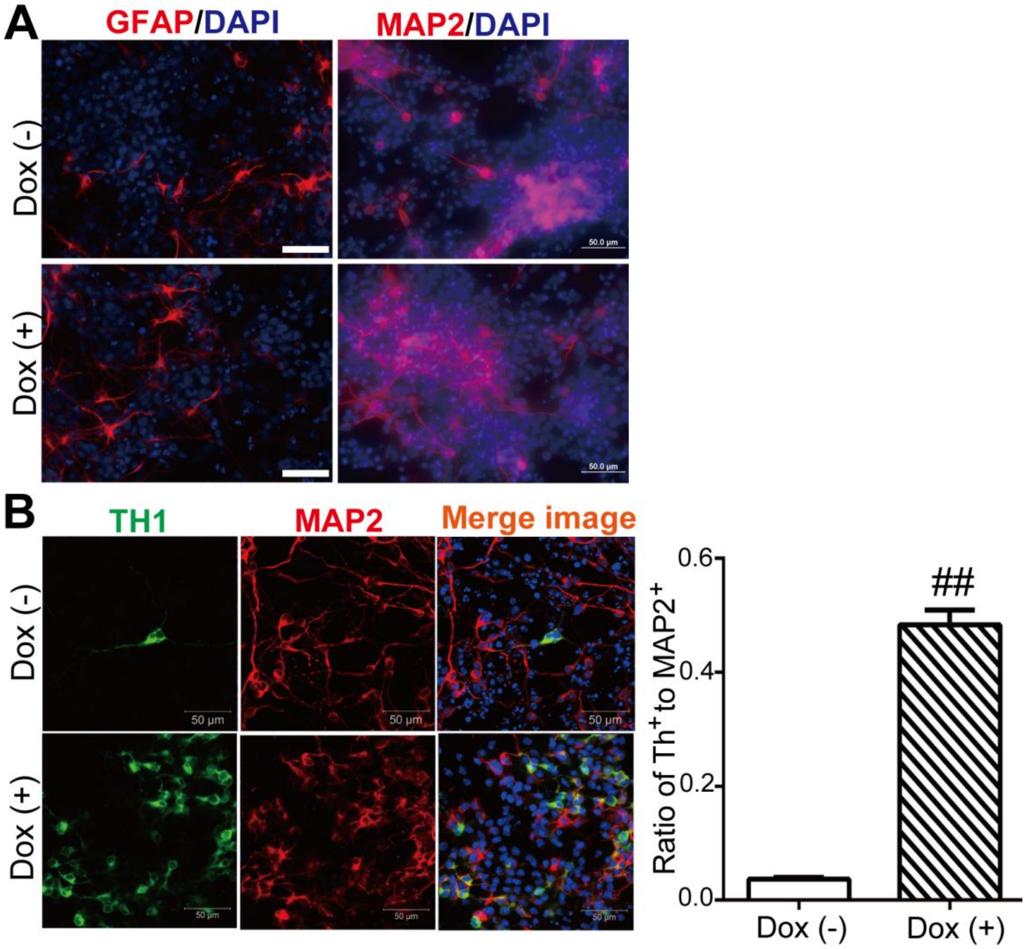

Supplemental figure 2: Overexpression of Lmx1α and Foxa2 increases the TH^{+} cell population, but has minimum effect on gliogenesis

5F-iNPCs with Dox-regulatable Lmx1α and Foxa2 were treated either with or without Dox under astrocyte differentiation condition (DMEM/F-12 supplied with 10% FBS) for

10 days, and neuronal differentiation condition (DMEM/F12 with N2, GDNF, and BDNF) for 14 days. The differentiations of neurons and astrocytes were visualized through immunofluorescence staining with MAP2 and GFAP, respectively (*A*). 5F-iNPCs with Dox-regulatable Lmx1α and Foxa2 were differentiated into DA neurons in the presence of GDNF, BDNF, AA and either with or without Dox for 8 days. The immunofluorescence staining of MAP2 (red) and TH (green) were carried out (*B*, *left panel*), and the ratio of TH^+ to $MAP2^+$ was quantified (mean ± SD) (*B*, *right panel*). * denotes $p < 0.05$. (Scale bar: 50 μm)

FIG.S3

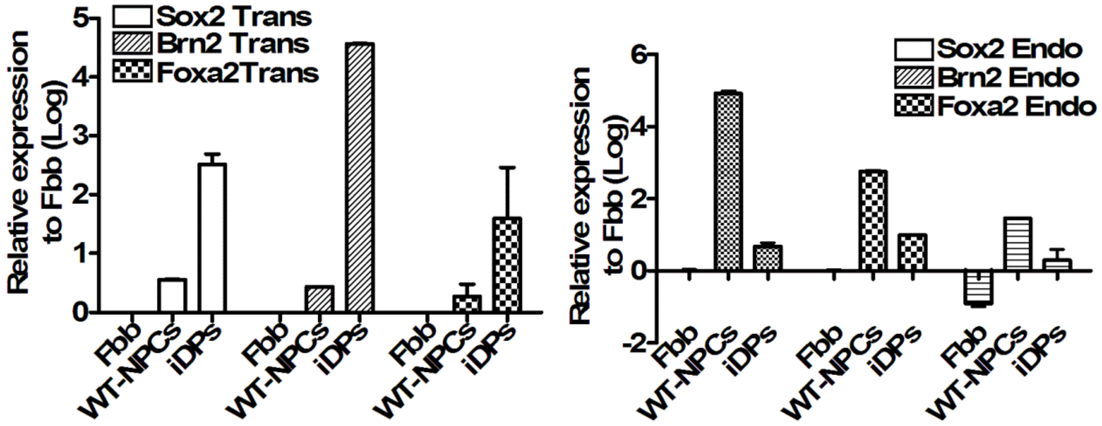

Supplemental figure 3: Analyses of transgenic and endogenous genes in iDPs

Real-time RT-PCR analysis of iDPs employing specific primer pairs for the detection of transgenic genes (*A*) and endogenous genes (*B*) used for iDP generation including Brn2, Sox2 and Foxa2. GAPDH-specific primer pairs were used for internal control; Fbb served as negative control and WT-NPCs served as positive control for endogenous detection.

FIG.S4

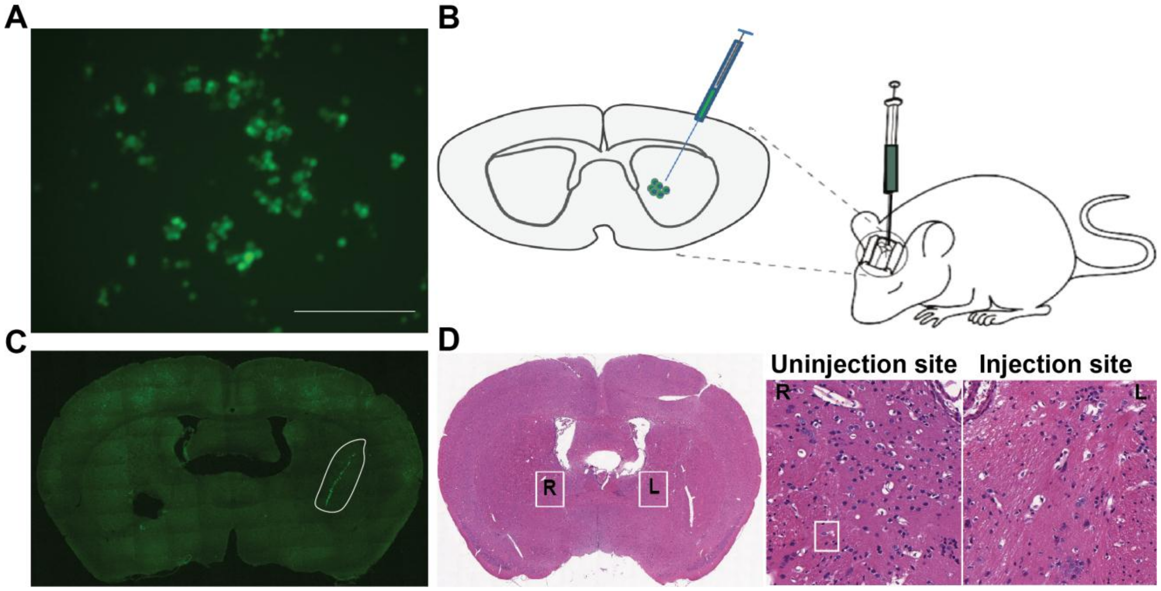

Supplemental figure 4: Cell injection, survival and safe evaluation of iDPs in SCID mice iDPs were infected with pLenti-CMV-GFP-Puroviruses and screened with puromycin (*A*). Schematic diagram of stereotactic injection of GFP-labeled iDPs in SCID mice (*B*). GFP-labeled iDPs were intracranially injected into the striatum of SCID mouse brain and images were captured using Leica confocal microscope and Metamorph analysis software (Molecular Devices) (*C*). Paraffin sections of formlin-fixed brain specimens were prepared 6 weeks after injection, and hematoxylin and eosin (H.E) staining shows tissues in injected (L, Left) and un-injected (R, Right) sides by Ventana's Coreo Au Slider Scanner (*D*)

Table S1. Primer pairs for SYBR-Green-based quantitative Real-Time RT-PCR

Genes	Forward Primers (5'→3')	Reverse Primers (5'→3')
Aldh1A1	GAGTGTGACGTGCTTCCAGA	TGGTCCCAGTTGATCATGGC
Pitx3	TGCGCTGTCGTTATCGGAC	GGTAGCGATTCCTCTGGAAGG
Lmx1α	ACGGCCTGAAGATGGAGGA	CAGAAACCTGTCCGAGATGAC
Nkx6-1	CTGCACAGTATGGCCGAGATG	CCGGGTTATGTGAGCCCAA
Msx1	TGCTGCTATGACTTCTTTGCC	GCTTCCTGTGATCGGCCAT
Corin	TGGAGGTGCCTATCAGAGAGA	GTGAGATCCAGTAACGCATTCA
Mash1	GCAACCGGGTCAAGTTGGT	GTCGTTGGAGTAGTTGGGGG
Ngn2	GACATTCCCGGACACACACC	CTCCTCGTCCTCCTCCTCGT
Foxg1	CAAGGCTGACGCACTTGGA	CTTGCCGTTCTTCTTGTCGC
Otx2	TATCTAAAGCAACCGCCTTACG	AAGTCCATACCCGAAGTGGTC
Gsx2	CATCATCAAGGACTCCTCACGG	GACATCACCAACGGGGACG
Nkx2-1	ATGAAGCGCCAGGCTAAGG	GGTTTGCCGTCTTTGACTAGG
GFAP	CTGGAACAGCAAAACAAGGCGCTGG	TCCAGCCTCAGGTTGGTTTCATC
Glast	ACCAAAAGCAACGGAGAAGAG	GGCATTCCGAAACAGGTAACTC
Nestin	CCCTGAAGTCGAGGAGCTG	CTGCTGCACCTCTAAGCGA
Olig1	TCTTCCACCGCATCCCTTCT	CCGAGTAGGGTAGGATAACTTCG
Olig2	TCCCCAGAACCCGATGATCTT	CGTGGACGAGGACACAGTC
Ng2	AGGGGTTCAGCTTTTCGGATT	AGTGTTATCATTCTCCGGGGTAG
S100β	TGGTTGCCCTCATTGATGTCT	CCCATCCCCATCTTCGTCC
Sox1	GAGATGATCAGCATGTACCTGCC	GTAGTGCTGTGGCAGCGAGT
Pax6	TGGCAAACAACCTGCCTATG	TGCACGAGTATGAGGAGGTCT
CD133	TGTTGTTGGCGCAAATGTGG	TGTTCCTTGAGCAGATAGGGA
Sox3	CAGCTCGAGAGAACGCATCA	ACGGGGTTCTTGAGTTCAGT
Zbtb16	CTGGGACTTTGTGCGATGTG	CGGTGGAAGAGGATCTCAAACA
L-myc	TTCTACGACTATGACTGCGGA	TGATGGAAGCATAATTCCTGCC
Brn2(endo)	AGCTGGAGAAGGAGGTGGTGAGAG	CACCTGCTACCTGATATAGGATAGTCCAGTG
Brn2 (tg)	AGCTGGAGAAGGAGGTGGTGAGAG	TTTATCGTCGACCACTGTGCTGG
Sox2 (endo)	CCTCCGGGACATGATCAGCATGTA	CGGCATCACGGTTTTTGCGT
Sox2 (tg)	CCTCCGGGACATGATCAGCATGTA	TTTATCGTCGACCACTGTGCTGG
Foxa2	TCAACCACCCCTTCTCTATCAACAACC	TGGGTAGTGCATGACCTGTTCGTAGG
Lmx1a (tg)	GATCCCTTCCGACAGGGTCTCAC	AGACTGCCTTGGGAAAAGCG
Foxa2 (tg)	TCAACCACCCCTTCTCTATCAACAACC	AGACTGCCTTGGGAAAAGCG

Table S2. 1st and 2nd antibodies used for immunofluorescence

1st Antibody	Isotype	Dilution	Source
GFAP	Rabbit Ig G	1:1,000	DAKO
MAP2	Mouse Ig G	1:1,000	Sigma
MAP2	Rabbit Ig G	1:1,000	Millipore
Nestin (10C2)	Mouse Ig G	1:1,000	ThermoFisher
O4	Mouse Ig G	1:1,000	R&D Systems
Synaptophysin	Rabbit Ig G	1:1,000	abcam
Msx GFP	Mouse Ig G	1:800	Millipore
Anti-Tyrosine Hydroxylase (TH)	Sheep Ig G	1:2,000	Jackson ImmunoResearch
Anti-Tyrosine Hydroxylase (TH)	Rabbit Ig G	1:1,000	Calbiochem®
βIII-Tubulin	Mouse Ig G	1:1,500	Sigma
βIII-Tubulin	Rabbit Ig G	1:1,500	Sigma
DOPA Decarboxylase	Rabbit Ig G	1:500	abcam
DAT	Rabbit Ig G	1:1,000	Biocompare
VMAT2 (9E11)	Mouse Ig G1	1:100	NOVUS Biologicals
Ki67	Rabbit Ig G	1:1,000	abcam
Corin (Lrp4)	Rabbit Ig G	1:1,000	R&D Systems
Doublecortin (DCX)	Goat Ig G	1:1,000	Santa Cruz Biotechnology
PSA-NCAM	Rabbit Ig G	1:1,000	Millipore
**2nd Antibodies**	**Isotype**	**Dilution**	**Source**
Alexa Fluor® 488	Goat anti-Rabbit Ig G	1:1,000	Molecular Probes
	Goat anti-Mouse Ig G	1:1,000	Molecular Probes
	Rabbit anti-Goat Ig G	1:1,000	Molecular Probes
	Rabbit anti-Mouse Ig	1:1,000	Molecular Probes
	Donkey anti-goat Ig G	1:1,000	Molecular Probes
	Donkey anti-Sheep Ig G	1:1,000	Jackson ImmunoResearch
Alexa Fluor® 594	Goat anti-Rabbit Ig G	1:1,000	Molecular Probes
	Donkey anti-goat Ig G	1:1,000	Molecular Probes
	Donkey anti-Rabbit Ig G	1:1,000	Molecular Probes
	Donkey anti-Sheep Ig G	1:1,000	Jackson ImmunoResearch
Alexa Fluor® 568	Goat anti-Mouse Ig G	1:1,000	Molecular Probes
Alexa Fluor® 647	Donkey anti-Rabbit Ig G	1:1,000	Molecular Probes
